# Clinical and genetic characterization of chanarin-dorfman syndrome patients: first report of large deletions in the ABHD5 gene

**DOI:** 10.1186/1750-1172-5-33

**Published:** 2010-12-01

**Authors:** Chiara Redaelli, Rosalind A Coleman, Laura Moro, Catherine Dacou-Voutetakis, Solaf Mohamed Elsayed, Daniele Prati, Agostino Colli, Donatella Mela, Roberto Colombo, Daniela Tavian

**Affiliations:** 1 Department of Psychology, Catholic University of the Sacred Heart, Milan, Italy; 2 Department of Nutrition, University of North Carolina, Chapel Hill, NC, USA; 3 DiSCAFF Department, University of Piemonte Orientale, Novara; 4 Department of Paediatrics, Athens University, Greece; 5 Medical Genetics Center, Korba, Cairo, Egypt; 6 Department of Transfusion Medicine and Hematology, Ospedale Alessandro Manzoni, Lecco, Italy; 7 Center of Transfusion Medicine, Cellular Therapy and CryoBiology, IRCCS Foundation Ca' Granda Ospedale Maggiore Policlinico, Milan, Italy; 8 Department of Internal Medicine, Ospedale Alessandro Manzoni, Lecco, Italy; 9 Department of Internal Medicine, Santa Corona Hospital, Pietra Ligure, Italy; 10 Institute of Biochemistry and Clinical Biochemistry, Catholic University, Gemelli Hospital, Rome, Italy

## Abstract

**Background:**

Chanarin-Dorfman syndrome (CDS) is a rare autosomal recessive disorder characterized by nonbullous congenital ichthyosiform erythroderma (NCIE) and an intracellular accumulation of triacylglycerol (TG) droplets in most tissues. The clinical phenotype involves multiple organs and systems, including liver, eyes, ears, skeletal muscle and central nervous system (CNS). Mutations in ABHD5/CGI58 gene are associated with CDS.

**Methods:**

Eight CDS patients belonging to six different families from Mediterranean countries were enrolled for genetic study. Molecular analysis of the ABHD5 gene included the sequencing of the 7 coding exons and of the putative 5' regulatory regions, as well as reverse transcript-polymerase chain reaction analysis and sequencing of normal and aberrant ABHD5 cDNAs.

**Results:**

Five different mutations were identified, four of which were novel, including two splice-site mutations (c.47+1G>A and c.960+5G>A) and two large deletions (c.898_*320del and c.662-1330_773+46del). All the reported mutations are predicted to be pathogenic because they lead to an early stop codon or a frameshift producing a premature termination of translation. While nonsense, missense, frameshift and splice-site mutations have been identified in CDS patients, large genomic deletions have not previously been described.

**Conclusions:**

These results emphasize the need for an efficient approach for genomic deletion screening to ensure an accurate molecular diagnosis of CDS. Moreover, in spite of intensive molecular screening, no mutations were identified in one patient with a confirmed clinical diagnosis of CDS, appointing to genetic heterogeneity of the syndrome.

## Introduction

Neutral-lipid storage diseases (NLSDs) are a clinically heterogeneous group of non-lysosomal inherited disorders characterized by the cytoplasmic accumulation of lipid droplets (LDs) in most tissues. Clinical phenotypes include myopathy (skeletal and heart muscle), liver damage, ataxia, neurosensory hearing loss, ichthyosis, sub-capsular cataracts, nystagmus, strabismus, and, rarely, mental retardation [1-6]. When non-bullous congenital ichthyosiform erythroderma (NCIE), presenting as fine scaling on erythematous skin, is the dominant feature of NLSD since birth, the disorder is referred to as Chanarin-Dorfman syndrome (CDS [MIM 275630]) or neutral lipid storage disease with ichthyosis (NLSDI). Patients are sometimes born as collodion babies. Serum lipids are normal, whereas muscle and hepatic enzymes are frequently elevated compared to control values. Besides granulocytes and other blood cells, LDs are seen in multiple cell types, including fibroblasts, basal keratinocytes, myocytes. and hepatocytes. Severe hepatic steatosis consequent to liver LD accumulation can cause life threatening portal hypertension [7,8]. Although ichthyosis is always present, other clinical features of CDS may vary. The biochemical defect found in all the CDS patients is attributed to deficient fatty acid (FA) mobilization [9-12]. As a consequence of this impairment, a number of tissues other than adipose accumulate TGs in LDs even in the absence of an excess of circulating FAs.

CDS is inherited as an autosomal recessive disorder and has been reported in approximately 55 cases, particularly in families whose origins are in the Mediterranean area and the Middle-East [13]. However, the presence of CDS Families from Saudi Arabia, India and Japan has also been reported [14]. The identification of mutations in ABHD5 gene (originally called CGI58 [UniGene Hs.19385]), located on chromosome 3p21, in nine CDS families from Algeria, Morocco, Turkey, and France (13) strengthened the suggestion that CDS is a unique clinical variety of NLSDs with a defined genetic cause. The ABDH5 gene is comprised of 7 exons that encompass about 28 kb of genomic DNA [MIM 604780]. The cDNA has an open reading frame of 1427 nucleotides that predict a 349-amino acid protein of approximately 39 kDa. ABDH5 has been reported to have two functions, one as a cofactor for adipose triglyceride lipase (ATGL) [15], and the other as a lysophosphatidic acid acyltransferase [16,17]. ATGL is a TG hydrolase that promotes the catabolism of stored fat in adipose and non adipose tissues [15]. The ATGL gene (alias PNPLA2) has been identified as the causative gene for the neutral lipid storage disease with myopathy without ichthyosis (NLSDM) [18].

To date, 22 point mutations and small insertions/deletions have been identified in the ABHD5 gene. We now report a molecular study of six additional CDS families from Southern Italy, Egypt, Palestine and Greece. Genetic analysis of ABHD5 coding regions and their flanking DNA sequences, as well as RT-PCR analysis of ABHD5 complete cDNA, allowed us to identify novel mutations, thus expanding the allelic spectrum of chromosome-3p21-linked CDS to large genomic deletions. The failure to detect any functional ABHD5 genomic variation in one family provides evidence for the genetic heterogeneity of CDS.

## Patients and methods

### Families and Specimens

Eight patients for whom a diagnosis of CDS had been unambiguously established and eight unaffected relatives from six families were investigated for ABHD5 mutations (Table 1). Two families were from Italy (Molise: patient A-II-1; Sicily: patient F-II-1) [7,19], one family from Egypt (patient B-II-1) [20], one from Palestine (patients C-II-2 and C-II-1) [21], one from Cyprus (patient D-II-2) [22] and one from Greece (Athens: patients E-II-1 and E-II-2) [23]. Two families were known to be consanguineous because of marriages between first cousins (Additional file 1 Figure S1). Signed, informed consent was obtained from each patient and each family member. Common diagnostic criteria were: congenital ichthyosiform erythroderma characterized by fine scales on an erythematous background, hepatomegaly or liver steatosis, raised serum levels of aminotransferases, and the presence of Jordans' bodies in granulocytes. The involvement of other organs and systems (spleen, eyes, ears, skeletal muscles, bone marrow, and CNS) was variable. Complete clinical evaluation of each CDS patient has been reported elsewhere (Table 1). Skin biopsies were obtained from three patients and two relatives, and fibroblast cultures were established in minimal essential medium with Earle's salts supplemented with 10% foetal bovine serum. The cells were subcultured by trypsinization, as required (5-15 passages), and aliquots were stored under liquid nitrogen. When fibroblast cultures were not available, DNA and RNA were extracted by conventional methods from whole blood samples drawn from patients and their relatives.

**Table 1:** Summary of Patients' clinical data

**Clinical features**	**Patients**							
	**A-II-1**	**B-II-1**	**C-II-2**	**C-II-1**	**D-II-2**	**E-II-1**	**E-II-2**	**F-II-1**
	
Age/sex	42 y (F)	1 y (M)	12 y (M)	13 y (M)	9 y (M)	8 y (M)	6 y (M)	16 y M)
Place of origin	Molise	Egypt	Palestine	Palestine	Greece-Cyprio	Greece	Greece	Sicily
Consanguinity	No	Yes	Yes	Yes	No	No	No	No
Lipid vacuoles in	Granulocytes and monocytes	Granulocytes, monocytes, skin, liver, bone marrow, epidermal Langerhans cells	Granulocytes, monocytes, skin	Granulocytes, monocytes, skin	Granulocytes, keratinocytes, fibroblasts, endothelial cells	Granulocytes, monocytes, skin	Granulocytes, monocytes, skin	Granulocytes, monocytes, skin, liver
Liver disease	Severe steatosis, splenomegaly, portal hypertension	Hepatosplenomegaly, steatosis	NE	NE	Hepatomegaly	Hepatomegaly, fatty infiltration, lobular fibrosis	Hepatomegaly, fatty infiltration, lobular fibrosis	Hepatomegaly
NCIE	Yes	Yes	Yes	Yes	Yes	Yes	Yes	Yes
Myopathy	No	Yes	Mild	Mild	Mild	No	No	No
Ophtalmological (Ophthalmologic examination)	Cataracts	Bilateral ectropion	Cataracts (Nuclear)	Cataracts (Nuclear)	microcataracts, myopia (Nuclear)	Cataracts	Cataracts	No
Deafness	Hypoacusia	No	Yes	Yes	No	No	No	No
CNS abnormalities	No	No	Neurological retardation	Neurological retardation	No	No	No	No
Altered biochemical analysis	AST, ALT	Triglycerides, AST	Serum muscle enzymes	Serum muscle enzymes	ALT, GGT, Serum muscle enzymes	AST, ALT, GGT	AST, ALT, GGT	GGT, transient increase of serum transaminases
Others	No	Umbilical hernia	Short stature, peculiar facial appearance	Mild lateral facial weakness	No	Short stature	No	Looking rather older than his age
Reported by	N. Ronchetti	Z. EI-Kabbany	M.L. Williams	M.L. Williams	M.R. Judge	T. Kakourou	T. Kakourou	D. Mela

y, years; m, months; NE: not examined; AST, aspartate aminotransferase; ALT, alanine aminotransferase; GGT, γ-glutamyl transpeptidase; CPK, creatine phosphokinase; SGOT, serum glutamic oxaloacetic transaminase; SGPT, serum glutamic pyruvic transaminase; CNS, central nervous system; NCIE, nonbullous congenital ichthyosiform erytrhoderma

### Cell microscopy

Peripheral blood smears or buffy coats were stained with the May-Grünwald-Giemsa and Nile Red (NR) stain to detect LDs in neutrophils and in monocytes by 100× light and fluorescence microscopy [24]. Fibroblasts were cultured on glass coverslips, allowed to adhere overnight, observed under phase-contrast light microscopy (40×; IX51, Olympus), fixed with 3.7% paraformaldeyde and stained with NR prior to fluorescence microscopy (40×). NR (Sigma-Aldrich) staining solution was freshly prepared in DPBS (1:100 v/v) from a saturated solution (1 mg/ml) in dimethylsulfoxide. Fluorescent images were captured using a Leica MB5000B microscope equipped with a DFC480 R2 digital camera and a Leica Application Suite (LAS) software.

### Genomic analysis

Oligonucleotides were selected to amplify and sequence the seven exons of ABHD5, their intron/exon boundaries, and the candidate promoter regions. The primer sequences are reported in Additional file 2 Table S1. PCR was performed in a 50 μl mixture containing 200 ng of genomic DNA, using a PTC-200 thermocycler (MJ Research). PCR conditions for genomic amplifications of exons 2-7 were reported by Lefèvre et al. [13] To amplify exon 1, we used the DyNAzyme EXT (Finnzymes), and the PCR reaction was performed in 10% DMSO (annealing: 50°C).

The sequence spanning 5 kb upstream from the ATG starting codon of the ABHD5 gene was scanned for transcription factors AP-2, Sp1, GCF, NF-D, T-Ag by the program PROSCAN Version 1.7. Two putative promoter sequences were identified through this analysis; they were localized at about 5 and 0.3 Kb upstream from the ATG starting codon of the ABHD5 gene, spanning respectively 251 and 245 nt. The first promoter region was amplified using 1F/1R primers, spanning from -313 nt of the ABDH5 promoter to 338 nt of ABDH5 exon 1. A nested PCR was performed, using aF/aR and bF/bR primer pairs, to analyze the second putative promoter region. The aF/aR cycling profile was as follows: denaturation at 94°C for 4 min, annealing at 50°C for 30 sec and extension at 70°C for 3 min for the first round; denaturation at 94°C for 30 sec, annealing at 50°C for 30 sec and extension at 70°C for 3 min for 30 cycles; denaturation at 94°C for 30 sec, annealing at 50°C for 30 sec and terminal extension at 70°C for 10 min for the last cycle. The PCR reaction was performed in 25 μl with 4% of DMSO, using the DyNAzyme EXT. 0.5 μl of aF/aR PCR was used to perform the next amplification with bF/bR primers. 30 cycles of amplification were performed using Taq Polymerase and the same cycling profile of aF/aR primers with the exception of the extension (45 sec).

ABHD5 large deletions were identified amplifying genomic DNA with 6F/7R and 4aF/6R primer pairs. PCR conditions for 4aF/6R primers were as follows: hot start at 96°C for 5 min; denaturation at 94°C for 40 sec, annealing at 55°C for 40 sec, extension at 68°C for 5 min for 30 cycles; denaturation at 94°C for 40 sec, annealing at 55°C for 40 sec and terminal extension at 68°C for 30 min for the last cycle. The JumpStart AccuTaq LA DNA Polymerase (Sigma) was used to perform the long-range PCR of this large fragment. All the PCR products were purified (NucleoSpin Extract II; M-Medical) and sequenced on 3730 DNA Analyzers (Applied Biosystems, Foster City, CA) by the BigDye Terminator V1.1 Cycle Sequencing Kit (Applied Biosystems).

### Reverse-Transcriptase PCR (RT-PCR) and cDNA analysis

Total RNA (1 μg) isolated from whole blood by the TRIzol method (Invitrogen, Carslbad, CA) was converted to cDNA by RT-PCR using random hexamers (0.5 μg), 400 units of MMLV-RT, 1.6 mM total dNTPs, 20 units of Rnasin, 0.4 mM dithiothreitol, in 50 μl of reaction solution containing 10 × RT Buffer. Five microliters of cDNA was used to perform PCR amplification using 8F/8R and 9F/9R primer pairs designed to produce overlapping fragments covering the entire sequence of the ABHD5 transcript (GenBank accession number AF151816). PCR conditions for 8F/8R primers were as follows: denaturation at 96°C for 3 min, annealing at 64°C for 40 sec and extension at 72°C for 1 min for the first round, denaturation at 95°C for 40 sec, annealing at 64°C for 40 sec and extension at 72°C for 1 min for 35 cycles; denaturation at 95°C for 40 sec, annealing at 64°C for 40 sec and terminal extension at 72°C for 3 min for the last cycle. For this PCR reaction the DyNAzyme EXT was used. PCR conditions for 9F/9R primer pairs consisted of an initial denaturation for 5 min at 94°C, 37 cycles of denaturation for 30 sec at 94°C, annealing for 30 sec at 56°C, and extension for 1 min at 72°C. Specific sets of primers were selected to reveal the consequences of ABHD5 nucleotide variations localized in splice-site sequences; they were 2aF/2R for c.47+1G>A and 9aF/9R for c.960+5G>A mutations. PCR conditions for 2aF/2R and 9aF/9R primer pairs were the same as those used for 9F/9R.

The PCR products were electrophoresed on a 2% agarose gel containing ethidium bromide and their sizes compared with those of the corresponding ABHD5 cDNA fragments from control subjects under a UV illuminator. RT-PCR products of CDS patients were sequenced.

## Results

### Patients

The two main clinical features of CDS (i.e. NCIE and hepatomegaly or liver steatosis) were present in all families investigated in this study, but with considerable variation in the extent and degree of organ involvement in individual patients (Table 1). Hepatosplenomegaly and steatosis were first observed in patient B-II-1 as early as 9 months of age and in D-II-2, E-II-1 and E-II-2 patients at age 20-22 months. In patients F-II-1 and A-II-1, hepatosplenomegaly was noted at age 16 and 42, respectively, when the patients were diagnosed clinically. Reduction in liver size was observed in patient E-II-1 (3 years old) after a medium-chain TG diet. At the age of 8 years, still on the special diet, the E-II-1 patient had a normal liver size and normal plasma aminotransferase activities [23]. Serum aminotransferases were moderately elevated in all patients, with the exception of C-II-2 e C-II-1. Pediatric-age cataracts were present in patients C-II-3, C-II-2, D-II-2, E-II-1 and E-II-2. On ophthalmologic examination, bilateral nuclear cataracts were also seen in A-II-1 at age 42. Bilateral ectropion was the only ocular abnormality in patient B-II-1. No ophthalmologic abnormalities were observed for F-II-1. As reported by other authors, clinical evidence of myopathy in NCIE patients usually begins in their thirties. Nevertheless, as shown by abnormal electromyography, one of our pediatric patients, B-II-1, presented with myopathy and C-II-2, C-II-1 and D-II-2 patients (13, 12 and 9 years old, respectively) presented with a mild myopathy. Serum creatine kinase was elevated in C-II-2, C-II-1 and D-II-2 patients, but was normal in B-II-1. Neurological abnormalities are generally considered to be a late manifestation of the disease. Most of our patients did not show any neurological impairment, with the exception of C-II-2 and C-II-1 patients. In these two patients, pure tone audiometry also demonstrated neurosensory deafness.

### MGG and fluorescence detection of LDs in leukocytes and fibroblasts from CDS patients

**Figure 1:**
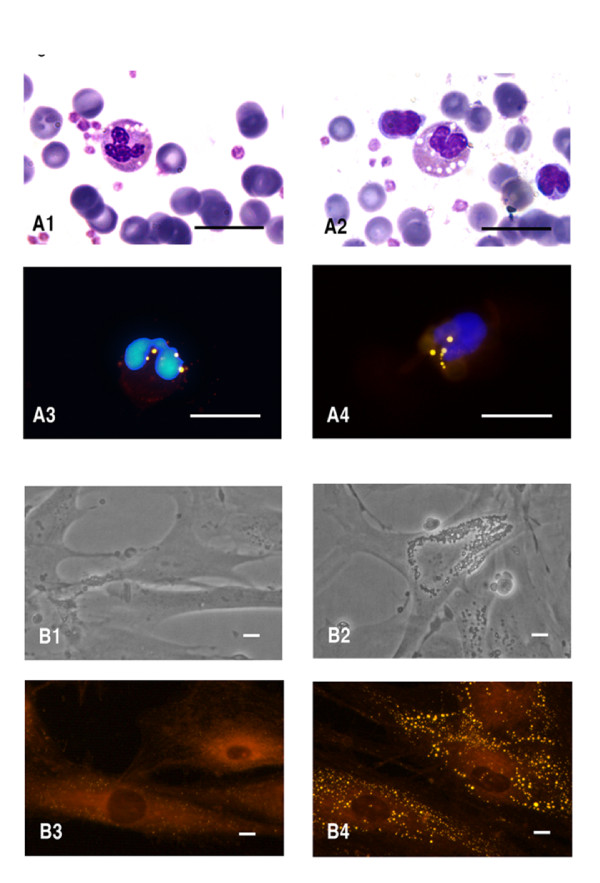
**Lipid droplets images obtained from CDS patients**. **A **Buffy coats from CDS patients; **A1,2 **Microphotographs of May-Grünwald-Giemsa and **A3,4 **of Nile red (NR) and DAPI-stained buffy coats. Scale bar: 10 μm. **B **Cultured fibroblasts from control (B1, B3) and affected (B2, B4) patients. Phase contrast images: B1,2. Fluorescent microscopy images with Nile red staining: B3,4. Scale bar: 40 μm.

In the peripheral buffy coat smears of all CDS patients, stained with MGG and NR, we identified a variable percentage of Jordans' bodies in neutrophilic and eosinophilic granulocytes and in monocytes (Figure 1A). Examination of peripheral blood smears demonstrated prominent cytoplasmic vacuoles in virtually every granulocyte and monocyte of C-II-2 and C-II-1 patients. Eighty to ninety percent of neutrophils, eosinophils and basophils contained multiple LDs, as did monocytes obtained from the remaining CDS patients (A-II-1, B-II-1, D-II-2, E-II-1 and E-II-2), with the exception of F-II-1, for whom lipid vacuoles were present in only 25% of granulocytes and monocytes. Semi-confluent CDS and control cultured fibroblasts were observed under phase-contrast light microscopy and stained with NR prior to fluorescence microscopy (Figure 1B). The number and size of LDs in CDS fibroblasts were consistently higher than in control cells. Large lipid vacuoles persisted through multiple passages and were present even when the CDS fibroblasts were grown in lipid-free media (data not shown).

### ABHD5/CGI58 Mutations

**Figure 2:**
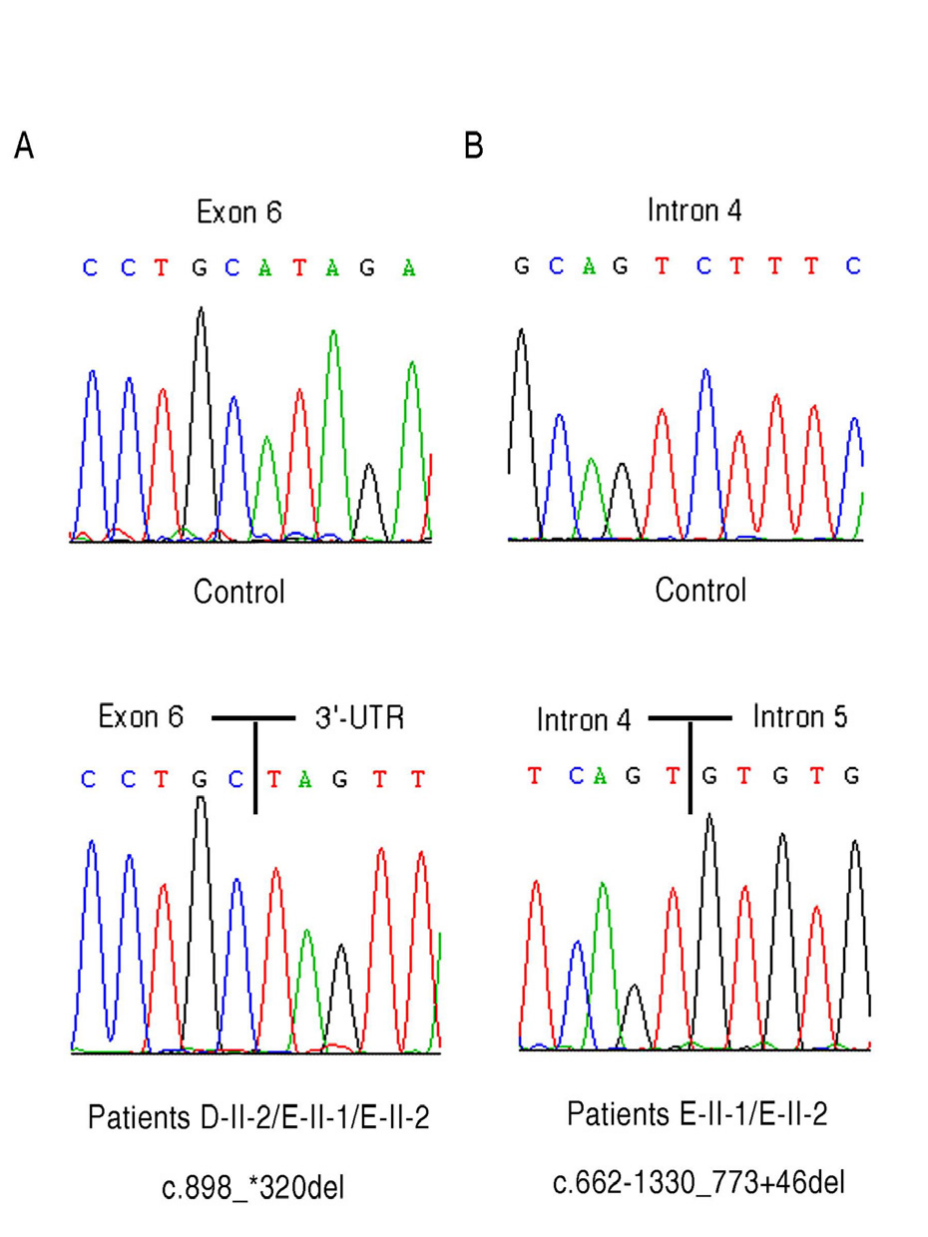
**Novel ABHD5 genomic rearrangements identified in CDS families**. **A **Sequence analysis showing c.898_*320del mutation. **B **Sequence analysis showing the c.662-1330_773+46del mutation.

**Figure 3:**
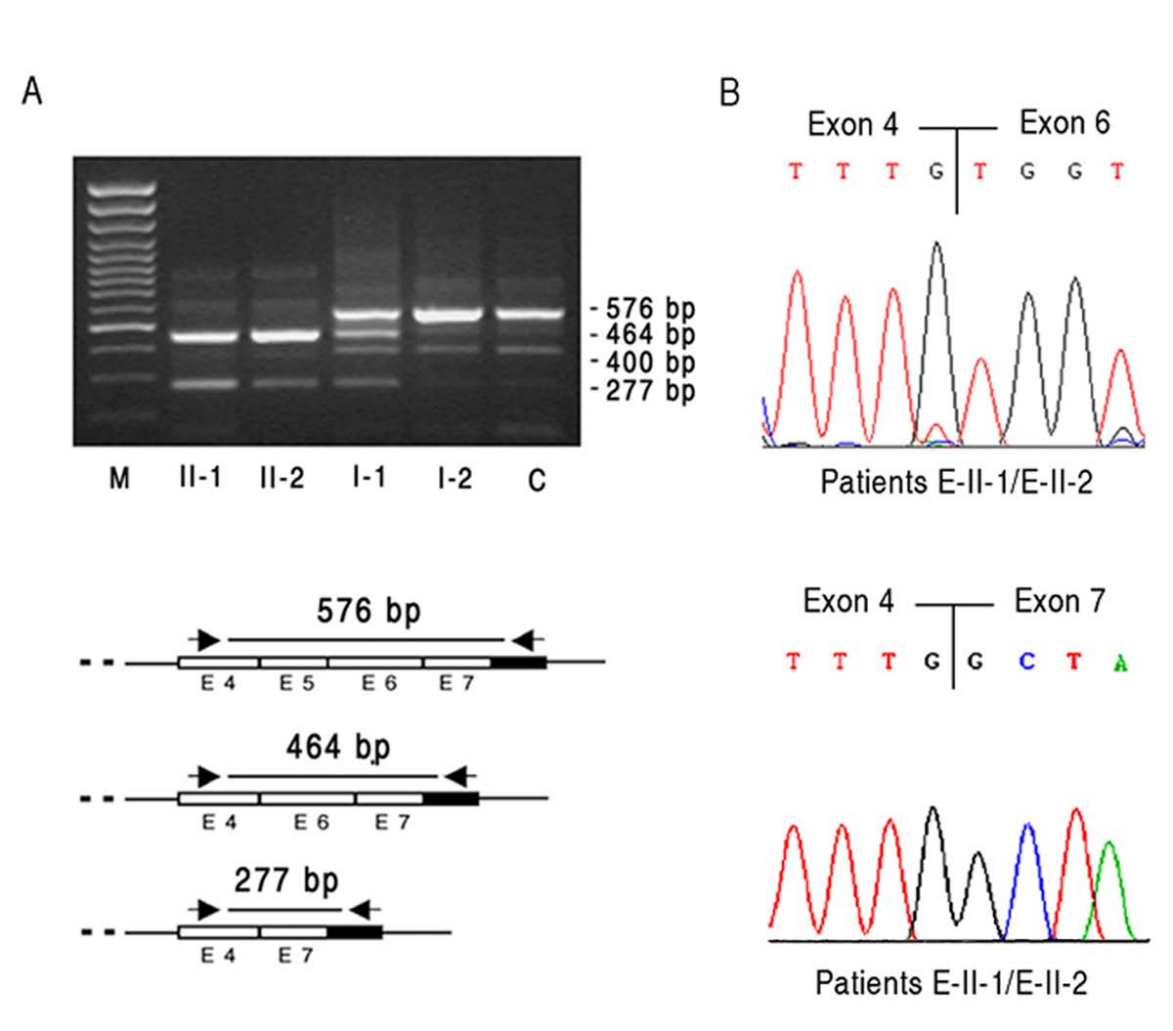
**Molecular characterization of the c.662-1330_773+46del in CDS family E**. **A**, RT-PCR performed with primers encompassing exons 4, 5, 6 and 7, showing absence of wild-type product (546 bp) in E-II-1 and E-II-2 patients and the presence of a dominant RT product of 464 bp and a minor product of 277 bp resulting from the skipping of exon 5 and of exons 5 and 6, respectively. Lane M: 100-bp molecular weight marker. Lanes II-1 and II-2: CDS patients. Lane I-1: father, carrying the c.662-1330_773+46del mutation in heterozygous state. Lane I-2: mother, carrying the other deletion. Lane C: control. **B**, Electropherograms of 464 bp and 277 bp abnormal RT-PCR products.

DNA sequence analysis of the putative promoter regions, of the seven coding exons and of the exon-intron boundaries of ABHD5 gene was performed in eight CDS patients and their relatives. Five different mutations were found in the six families from the Mediterranean area (Table 2). 5 CDS patients, A-II-1, B-II-1, C-II-2, C-II-1 and D-II-2, were homozygotes for ABHD5 mutations, and two patients, E-II-1 and E-II-2, were compound heterozygotes. No ABHD5 mutations were identified in patient, F-II-1. The new sequences were submitted to GenBank (accession numbers are shown in Table 2). All identified mutations segregated within families. The wild-type ABHD5 genomic sequence was extracted from GenBank accession number NG_007090.3. In the two families of Greek (E) and Greek-Cypriot origin (D), two new genomic rearrangements were detected. Patient D-II-2 showed the c.898_*320del mutation resulting in premature termination of the protein, p.I300X (Additional file 3 Figure S2). Sequence analysis revealed a 1058 deletion that removed 63 bp of exon 6, intron 6 and exon 7 (Figure 2A). Although the D and E CDS families are not known to be related, we found the same c.898_*320del mutation in E-II-1 and E-II-2 patients, providing evidence for the existence of a distal common ancestor. E-II-1 and E-II-2 subjects inherited this deletion maternally and another new large deletion paternally, the c.662-1330_773+46del mutation (Figure 2B). The last rearrangement occurred within intron 4 and removed part of intron 4, exon 5 and part of intron 5. ABHD5 cDNA encompassing exons 4, 5, 6 and 7 was examined by RT-PCR primers in control, heterozygous parents (E-I-1, E-I-2) and patients' samples (E-II-1, E-II-2) (Figure 3A). Normal (576 bp) and aberrant PCR products (464 and 277 bp) were excised and sequenced, showing that one aberrant band resulted from the exon 5 skipping and the second one from skipping of both exons 5 and 6 (Figure 3B). The ABHD5 mRNA lacking exon 5 was the most common transcript in E-II-1 and E-II-2 patients. The aberrantly spliced mRNA would be expected to result in the production of a protein lacking 129 amino acids in the C-terminal region of ABHD5 (p.G221VfsX9). Another RT-PCR product of about 400 bp was present in E-II-1, E-II-2 and control subjects; it consisted of non-specific sequence (Figure 3A).

**Table 2:** ABHD5 gene mutations

Patients	DNA position	cDNA o DNA mutation	Protein mutation	**GenBank accession number**^**a**^
**A-II-1**	**IVS 1**	**c.47+1G>A**	**p.S17fsX1**	HM474790
B-II-1	E 5	c.700C>T	p.R234X	/
**C-II-2**	**IVS 6**	**c.960+5G>A**	**p.A321VfsX10**	HM474791
**C-II-1**	**IVS 6**	**c.960+5G>A**	**p.A321VfsX10**	HM474791
**D-II-2**	**E 6/IVS 6/E 7**	c.898_*320del	**p.I300X**	HM474793
**E-II-I**	**E 6/IVS 6/E 7** **IVS 4/E 5/IVS 5**	c.[898_*320del]+ **[662-1330_773+46del]**	**p.[I300X]+** **[G221VfsX9]**	HM474793; HM474792
**E-II-2**	**E 6/IVS 6/E 7** **IVS 4/E 5/IVS 5**	c.[898_*320del]+ **[662-1330_773+46del]**	**p.[I300X]+** **[G221VfsX9]**	HM474793; HM474792

cDNA numbering begins with +1 as the A of the translation initiation codon; the translation initiator methionine is numbered as +1; novel mutations are typed in bold; E, exon; IVS, intervening sequence; c, cDNA; g, gene.

^a ^GenBank accession number of the new mutations identified in this study

In the CDS patient from Molise, A-II-1, a novel homozygous mutation, the c.47+1G>A, was detected. This is a splice-site mutation affecting the invariant G of the intron-1 donor splice-site GT dinucleotide (Additional file 4 Figure S3A). This splice-site mutation is expected to lead to aberrant splicing with retention of intron 1; this was confirmed by RT-PCR (Additional file 5 Figure S4A). Using the 2F/2aR primer pairs, no amplification product was expected from control cDNA since the forward primer (2F) localizes within intron 1. A 215 bp RT-PCR product was obtained from A-II-1 cDNA amplified with the 2F/2aR primers, showing that intron 1 was not eliminated during RNA splicing in this patient. Direct sequencing of the 215 bp RT-PCR product confirmed that A-II-1 cDNA contained the entire intron 1 sequence (Additional file 5 Figure S4C). The c.47+1G>A mutation resulted in a truncation of the ABHD5 ORF at a premature stop codon located at the beginning of intron 1. The mutated protein is predicted to consist of only 17 amino acids (pS17fsX1).

A novel mutation was also detected in the C-II-2 and C-II-1 patients from Palestine. This homozygous splice-donor-site mutation, c.960+5G>A, caused abnormal RNA splicing (Additional file 4 Figure S3B). The region of the ABHD5 transcript, including exons 6 and 7, was amplified by RT-PCR. Instead of the expected cDNA fragment (236 bp), a longer cDNA fragment of 821 bp in size was detected (Additional file 5 Figure S4B). Sequence analysis showed that the longer cDNA product arose from abnormal retention of intron 6 (Additional file 5 Figure S4D). The c.960+5G>A splice-site mutation creates a premature stop codon within intron 6 and removes 29 amino acids from the C-terminal tail of the protein (p.A321VfsX10).

In the Egyptian family, the p.R234X mutation was present. The same mutation had been identified in an adult case of CDS by Schleinitz et al. [25]. None of the novel mutations, identified in CDS patients, were observed in more than 100 alleles from control subjects. Finally, RT-PCR analysis and sequencing of full-length ABHD5 cDNA was performed in F-II-1 patient (Sicily) for which no genomic mutations were found. This result confirmed the negative DNA analysis and excluded distant intronic mutations that might have affected mRNA splicing. Furthermore, direct sequencing of two putative promoter regions upstream the ATG starting codon of ABHD5 gene failed to detect any pathogenic mutation.

## Discussion

Since the identification of the ABHD5/CGI58 gene and the detection of its mutations in nine CDS families by Lefèvre et al [13], CDS (NLSD with ichthyosis) has been considered to be a unique clinical variant of NLSD with a defined genetic cause. ABHD5 is a co-activator of ATGL, a novel lipase that catalyses the initial step of TG hydrolysis in adipocyte and non-adipocyte LDs [15]. These data suggest an important biochemical role for ABHD5 in the intracellular catabolism of neutral lipids and provide an explanation for the pathogenic effects of ABHD5 mutations. In addition, ABHD5 is a lysophosphatidic acid acyltransferase; the relationship of this activity to the clinical phenotype remains unclear [16,17].

**Figure 4:**
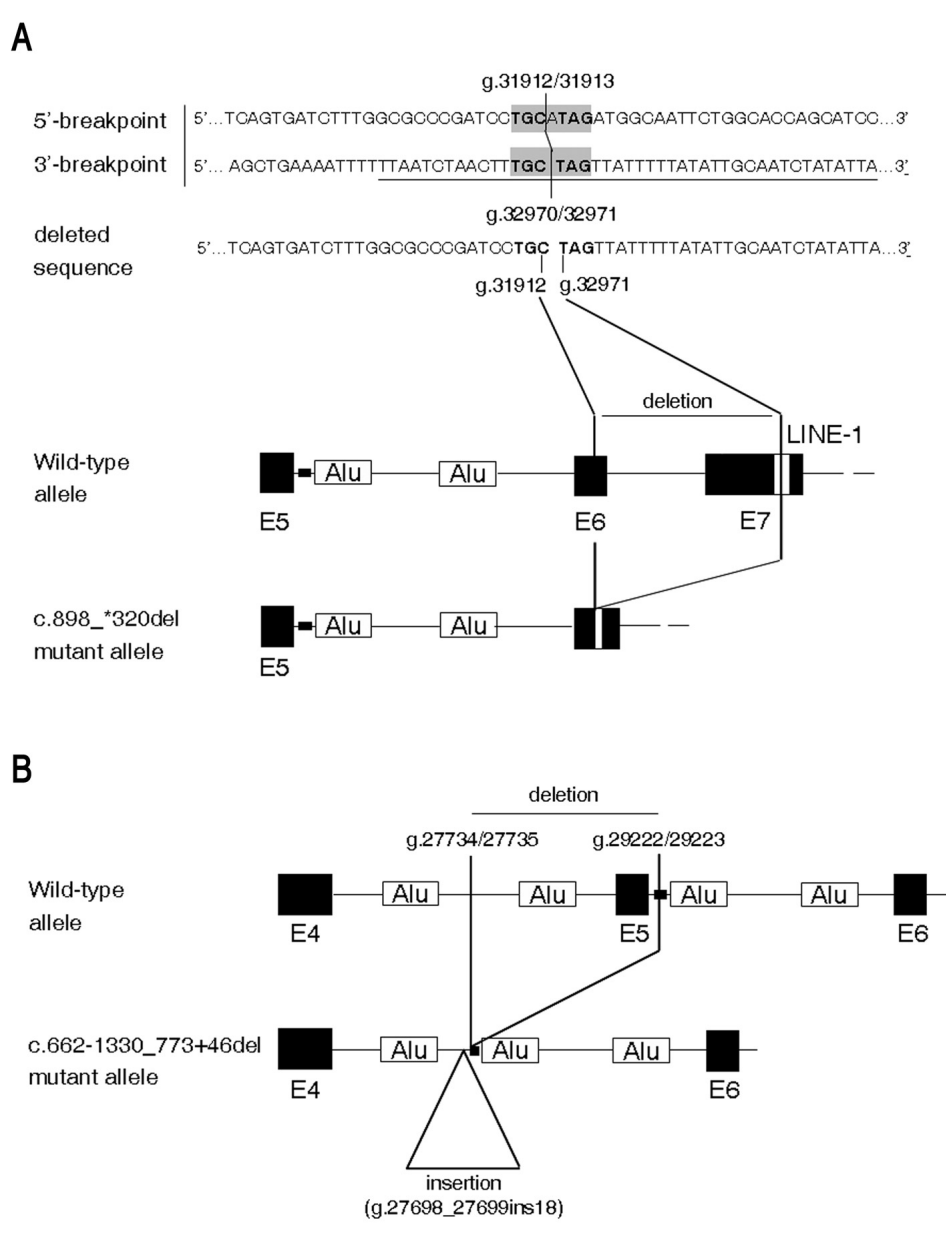
**Diagram of the two large deletions identified in CDS-D and CDS-E families**. **A**, Diagram of the 1058 bp deletion found in D-II-1, E-II-2 and E-II-1 patients. Normal sequences at the 5' and 3' breakpoints of the deletion are aligned with the deleted sequence. The 6 bp micro-homology at the breakpoints is highlighted in grey. Part of the Alu sequence at the 3'-breakpoint is underlined. The structure of the abnormal allele in the region of the deletion is shown at the bottom of the diagram. **B**, Diagram of the 1487 bp deletion identified in E-II-2 and E-II-1 patients. The breakpoints, reported on the model, are inside intron 4 and inside a GT repeat (black area) in intron 5. The site of the 18 bp insertion is also reported. It is in very close proximity of the breakpoint.

Our study extends the spectrum of ABHD5 disease-causing mutations in CDS. Sequence analysis reveals five different mutations distributed all along the ABHD5 gene in eight CDS patients from six families from the Mediterranean area. The identified genetic variations include one nonsense mutation, two splice-site substitutions and, for the first time, two large deletions. In order to verify whether these large genomic rearrangements could be explained by a common mutational mechanism, the RepeatMasker Software (Institute for System Biology, Seattle, WA, USA) was used and the repeat elements or sequence homologies around the breakpoints were analyzed. While the proximal breakpoint of c.898_*320del mutation (g.31913_32970del1058) is not located within any repeat sequence, its distal breakpoint lays within a X7B LINE retrotransposon which belongs to the long interspersed elements LINE-1 (or L1) (g.32956_33063). Moreover, the presence of a micro-homology of 3 bp plus 3 bp (i.e. TGC and TAG) in the junction sequence/breakpoints accounts for the model of replication slippage [26] (Figure 4A). The second large deletion c.662-1330_773+46del (g.27735_29222del1487) is also associated in *cis *to a 18 bp (g.27698_27699ins18) insertion and a nucleotide deletion (g.27730delG), which are not reported as ABHD5 polymorphisms (Figure 4B). The c.662-1330_773+46del proximal breakpoint is near an Alu sequence, while its distal breakpoint is located within a TG-simple repeat and very close to an Alu element (distance: 75 bp). Inside the deleted region there is another Alu sequence which is lost in the mutated allele. Alu repeat density in this region of ABHD5 gene (27010-29611) is very high. These Alu elements belong to the Alu-Sx subfamily and, like other repetitive sequences, have been shown to be involved in molecular mechanisms leading to rearrangements in the human genome [27]. The data show that the c.662-1330_773+46del mutation should be considered as a complex gene rearrangement. This is probably due to a different mutational mechanism in comparison with that which occurred for the c.898_*320del mutation. The consequences of gene deletions have been investigated through an exhaustive analysis of normal and aberrant ABHD5 mRNAs. These deletions may lead to shorter ABHD5 proteins, pG221VfsX9 and pI300X, lacking 139 and 50 COOH-terminal amino acids, respectively.

The splicing errors identified in our CDS families all represent novel mutations. They are homozygous G to A transitions which occurred in the splice consensus motifs of introns 1 and 6. In patient A-II-1 from Molise, the c.47+1G>A mutation eliminates approximately 96% of the ABHD5 protein. The consequence of this mutation may lead to the complete absence of the mutated protein, through protein instability. The E-II-2 and E-II-3 patients of Palestinian origin presented with the c.960+5G>A mutation. This mutation is expected to give rise to a truncated protein lacking 28 amino acids at the C-terminal region of ABHD5 (consisting of 320 out of 349 amino acids).

In the Egyptian patient (B-II-1), the R234X homozygous mutation conserves 67% of the ABHD5 wild-type protein. This patient was a one-year-old boy with generalized ichthyosis, bilateral ectropion, hepatosplenomegaly (noted at the age of 9 months), myopathy and an umbilical hernia. The same mutation had been previously identified in a 42-year-old man from France, who was heterozygous for R284X and H82R [25]. Despite delayed confirmation of the diagnosis, this patient presented with typical symptoms of CDS, including NCIE, muscle weakness, bilateral sub-capsular cataracts and neurosensory hearing loss. He did not have liver dysfunction or CNS abnormalities. Some important clinical differences emerged between the Egyptian and French patients, concerning, in particular, the hepatic involvement. These differences might be due to homozygous versus heterozygous conditions or by modifier genes and epigenetic factors which might be involved in these variations.

All the mutations described in this work are predicted to result in truncated proteins that lack different ABHD5 regions (Additional file 6 Figure S5). In spite of our efforts, it remains very difficult to find a correlation between the phenotypic and genotypic characteristics, since most of ABHD5 mutations are novel and unique. Surprisingly, we noticed that the c.960+5G→A mutation, which conserves 92% of the native protein, was associated with patients severely affected by neurological symptoms, hearing loss and myopathy (C-II-2, C-II3), whereas the c.47+1G>A mutation that caused a dramatic truncation of the ABHD5 protein (p.S17X), was associated with severe steatohepatitis but a relatively mild phenotype concerning the other clinical CDS features (patient: A-II-1). It is possible that the accumulation of non-functional ABHD5 proteins has a more deleterious consequence for cellular LD metabolism in some tissues than total loss of the protein expression. However, any explanation of this phenomenon remains highly speculative, since the precise roles of the ABHD5 domains remain largely unknown. Nevertheless, we can postulate that the consequence of the c.47+1G>A mutation (p.S17X) would be similar to classical loss of function or a knockout mutation, as the residual peptide contains only 17 of the 349 amino acids of the wild-type protein.

The N-terminal region ABHD5 (1-30 amino acids) is essential for correct localization to the lipid droplet and ABHD5 lacking this amino acid region, loses the ability to activate ATGL [28]. Four of the protein variants retain the hydrophobic motif (Additional file 6: Figure S5) but lack the HX_4_D motif, between amino acids 327 and 332, specific for proteins with acyltransferase activity [16]. Moreover, only 2 mutants retain both Q130 and E260, amino acid residues were previously identified as essential for ABHD5-perilipin interaction as well as for ATGL activation [15,29]. ABHD5 is a binding partner for perilipin and ADRP, two proteins of the PAT-domain family associated with the surface of LDs. Using double-label immunocytochemistry, Granneman et al. [30] found that perilipin acts as a link for ABHD5 when adipocytes are in the basal state. After protein kinase A (PKA) phosphorylation, perilipin decreases its interaction with ABHD5 and ABHD5 increases its co-localization with ATGL. One of the most important issues that remains to be solved is to identify ABHD5 regions (epitopes) that specifically interact with perilipin or with other proteins. Structure and function analysis of mutated ABHD5 proteins should be performed in order to investigate potential intra-molecular interactions between epitopes, as well as binding affinity to LDs proteins.

We were unable to identify any mutation in ABHD5 in patient F-II-1. When this patient was first examined at the age of 16, his health was good, although he had congenital ichthyosis, hepatomegaly, a persistent increase of GGT and a transient increase of transaminase, with no history of drug or alcohol abuse. On the peripheral blood smear, LDs were detected in only 20-30% of neutrophilic and eosinophilic granulocytes and in monocytes. On the basis of the clinical and histological findings, F-II-1 was diagnosed as having CDS [19]. To provide an exhaustive analysis of the ABHD5 gene in this patient, we screened the putative promoter/regulatory regions but failed to find any variation. The absence of any mutation in the ABHD5 gene in F-II-1 suggests that the etiology of CDS is genetically heterogeneous. Similar to our results, in one family of Algerian origin presenting with a CDS phenotype, Lefèvre et al. [13] identified two regions of homozygosity, one consisting of 11 cM on chromosome 3p21 (containing the ABHD5 gene), and the other spanning 18 cM on chromosome 14; this report and our evidence point to possible genetic heterogeneity of the syndrome. Although the F-II-1 patient had no evidence of myopathy, we also sequenced the ATGL gene but found no mutation.

CDS arises from a defect of LD metabolism leading to a systemic increase in the size and number of these cytosolic inclusions. Despite their classic denomination as simple lipid structures, LDs are complex and highly dynamic organelles [31] with a large complement of associated proteins, including scaffold proteins, lipases and co-activators, which have been found to change their associations with lipid droplets in response to lipolytic stimulation. Relatively little is known about temporal and spatial relationships among these LD proteins. Further studies are needed to identify new candidate genes involved in NLSDs, and further extensive ABHD5 gene analysis in a larger panel of CDS families would be useful in order to gain additional insights into the variability of clinical expression and the factors contributing to CDS.

## Conclusions

Our ABHD5 mutational analysis extends the molecular genetic heterogeneity of CDS. We have identified new splice site mutations and, for the first time, novel large deletions, demonstrating that sequencing, long range PCR and RT-PCR analysis are necessary to perform a complete molecular screening for ABHD5 gene mutations in order to avoid allelic drop-out phenomena. Moreover, our findings contribute to understanding of the complex effects that different ABHD5 gene mutations may have on the CDS phenotype.

## Abbreviations

CDS: Chanarin-Dorfman syndrome; CNS: central nervous system; FA: fatty acid; GGT: γ-glutamyl transpeptidase; LD: lipid droplet; MGG: May-Grünwald-Giemsa; NCIE: nonbullous congenital ichthyosiform erythroderma; NLSDs: Neutral-lipid storage diseases; NR: Nile Red; PKA: protein kinase A; TG: triacylglycerol.

## Competing interests

The authors declare that they have no competing interests.

## Authors' contributions

CR carried out the molecular genetic studies and the interpretation of the results. RAC and LM made substantial contributions to interpretation of data and participated in manuscript preparation. CDV, SME, DP, AC and RC were involved in the clinical evaluation of patients and manuscript revision. DT made substantial contributions to conception, analysis and interpretation of data and drafted the manuscript. All authors read and approved the final manuscript.

## Supplementary Material

Additional file 1 **Supplementary Figure 1**. Pedigrees of the CDS families. Click here for file

Additional file 2 **Supplementary Table 1**. Primers for genomic and cDNA analysis of ABHD5 gene. Click here for file

Additional file 3 **Supplementary Figure 2**. PCR products obtained utilizing 6F/7R primers in a control and the D-II-2 patient. While an expected band of 1338 bp was present in the control sample, a 280 bp product was detected in the CDS patient. The shorter PCR product differs from control for about 1050 bp. Click here for file

Additional file 4 **Supplementary Figure 3**. ABHD5 novel splice-site mutations identified in the A and C CDS families. **A**, mutation affecting the invariant G of the donor splice-site of intron 1 (c.47+1G>A) in A-II-1. **B**, mutation in the conserved donor splice-site of intron 6 (c.960+5G>A) in C-II-2 and C-II-3. Arrowheads indicate the positions of the mutations in affected patients. Lane 1: 100-bp molecular weight marker. Click here for file

Additonal file 5 **Supplementary Figure 4**. Molecular characterization of the c.47+1G>A and c.960+5G>A ABHD5 mutations. **A**, RT-PCR of part of intron 1 and exon 2 from cDNA of the control subject (no amplification product) and A-II-1 patient (215 bp); Lane 1: 100-bp molecular weight marker. **B**, RT-PCR of exons 6 and 7 from cDNA of a control subject (236 bp) and C-II-2 or C-II-3 patient (821 bp); Lane 1: 100-bp molecular weight marker. **C**, Partial sequences of exon1/exon2 from cDNA of a control subject and of intron1/exon2 from cDNA of the A-II-1 patient. **D**, Partial sequences of exon6/7 from cDNA of a control subject and of exon6/intron6 from cDNA of the C-II-2 or C-II-3 patients. Click here for file

Additional file 6 **Supplementary Figure 5**. Domain organization of wild-type and mutant ABHD5 variants. The ABHD5 theoretical variants resulting from the six mutations identified in this study are truncated proteins lacking different portions of wild-type ABHD5. Five of the six mutant variants retained the hydrophobic motif, located between residues 69 and 87 (dark-grey area), that represents the putative lipid-binding domain. However, all six mutant proteins lacked the HX_4_D motif between amino acids 327 and 332, specific for proteins with acyltransferase activity. Q130 and E260, reported in the models, have previously been identified as essential residues for ABHD5-perilipin interaction and for ATGL activation. Click here for file
