# Correction:Clinical and genetic characterization of chanarin-dorfman syndrome patients: first report of large deletions in the ABHD5 gene

**DOI:** 10.1186/1750-1172-6-6

**Published:** 2011-02-21

**Authors:** Chiara Redaelli, Rosalind A Coleman, Laura Moro, Amalia Sertedaki, Talia Kakourou, Solaf Mohamed Elsayed, Daniele Prati, Agostino Colli, Donatella Mela, Roberto Colombo, Daniela Tavian

**Affiliations:** 1 Department of Psychology, Catholic University of the Sacred Heart, Milan, Italy; 2 Department of Nutrition, University of North Carolina, Chapel Hill, NC, USA; 3 DiSCAFF Department, University of Piemonte Orientale, Novara, Italy; 4 Department of Paediatrics, Athens University, Greece; 5 Medical Genetics Center, Korba, Cairo, Egypt; 6 Department of Transfusion Medicine and Hematology, Ospedale Alessandro Manzoni, Lecco, Italy; 7 Center of Transfusion Medicine, Cellular Therapy and CryoBiology, IRCCS Foundation Ca' Granda Ospedale Maggiore Policlinico, Milan, Italy; 8 Department of Internal Medicine, Ospedale Alessandro Manzoni, Lecco, Italy; 9 Department of Internal Medicine, Santa Corona Hospital, Pietra Ligure, Italy; 10 Institute of Biochemistry and Clinical Biochemistry, Catholic University, Gemelli Hospital, Rome, Italy

## Correction

Following the publication of this article [1], it was clarified that the clinical follow-up of one of CDS family described in the manuscript was performed by Dr. Amalia Sertedaki and Talia Kakourou. The authorship of the article has been changed accordingly. The submitting authors would like to apologise to Amalia Sertedaki and Talia Kakourou for this error and they would like to thank Catherine Dacou-Voutetakis for underlining the problem.

## Competing interests

The authors declare that they have no competing interests.

## Authors' contributions

CR carried out the molecular genetic studies and the interpretation of the results. RAC and LM made substantial contributions to interpretation of data and participated in manuscript preparation. AS, TK, SME, DP, AC and RC were involved in the clinical evaluation of patients and manuscript revision. DT made substantial contributions to conception, analysis and interpretation of data and drafted the manuscript. All authors read and approved the final manuscript.
