# Supplementary figures and images for: Clinical and genetic characterization of chanarin-dorfman syndrome patients: first report of large deletions in the ABHD5 gene
Source: Orphanet J Rare Dis. 2010 Dec 1;5:33. doi: 10.1186/1750-1172-5-33 (PMC3019207; doi:10.1186/1750-1172-5-33)

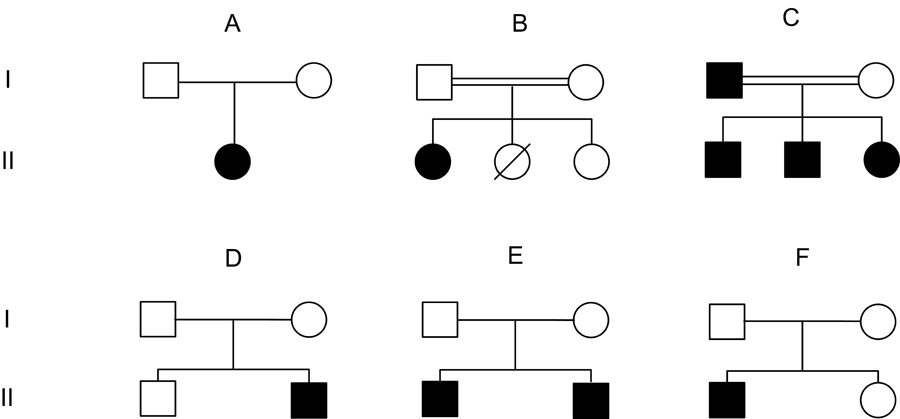

Supplement: Additional file 1 — Supplementary Figure 1. Pedigrees of the CDS families. [file 1750-1172-5-33-S1.JPEG]

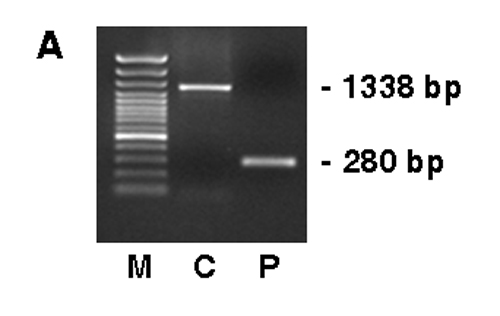

Supplement: Additional file 3 — Supplementary Figure 2. PCR products obtained utilizing 6F/7R primers in a control and the D-II-2 patient. While an expected band of 1338 bp was present in the control sample, a 280 bp product was detected in the CDS patient. The shorter PCR product differs from control for about 1050 bp. [file 1750-1172-5-33-S3.JPEG]

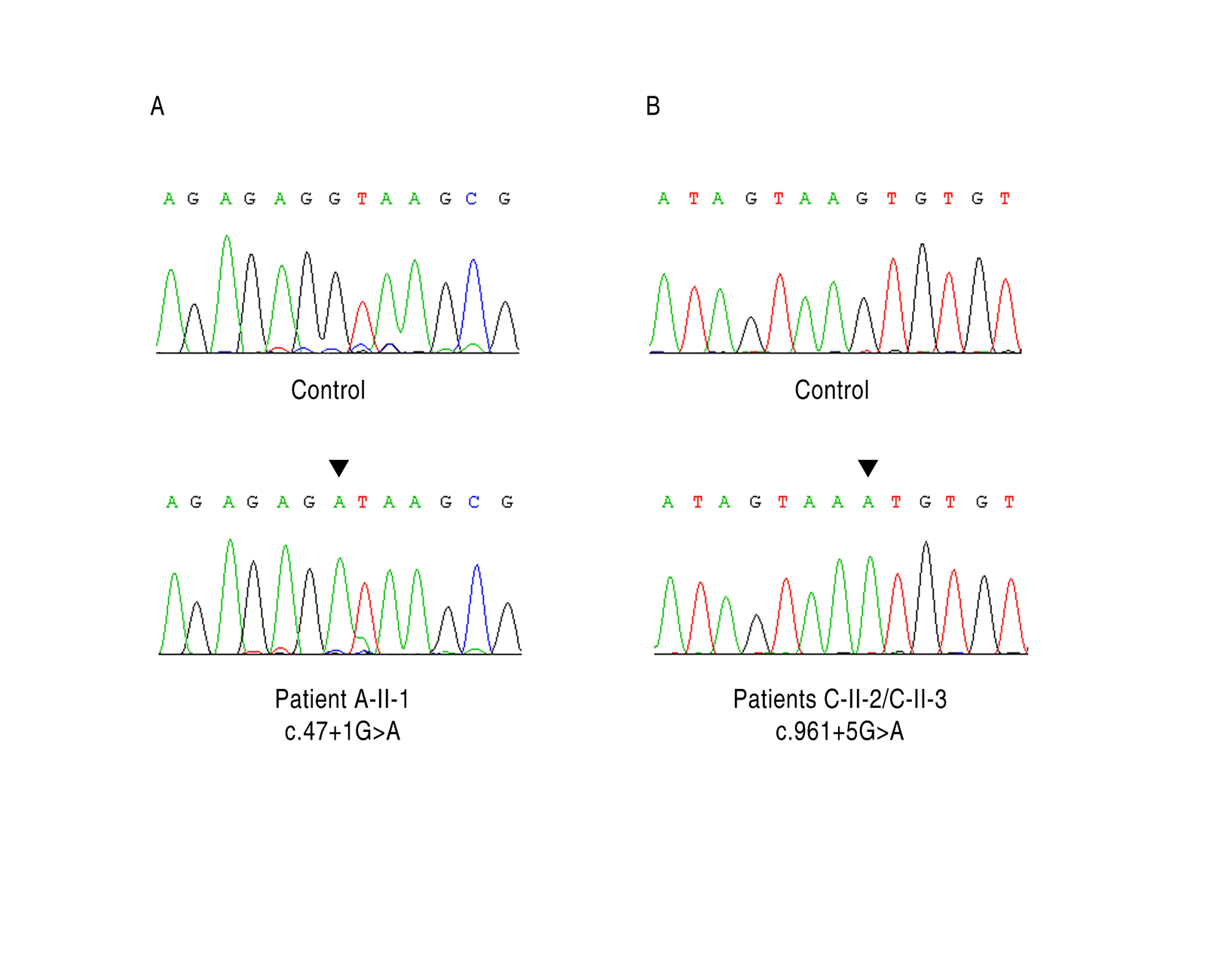

Supplement: Additional file 4 — Supplementary Figure 3. ABHD5 novel splice-site mutations identified in the A and C CDS families. A, mutation affecting the invariant G of the donor splice-site of intron 1 (c.47+1G>A) in A-II-1. B, mutation in the conserved donor splice-site of intron 6 (c.960+5G>A) in C-II-2 and C-II-3. Arrowheads indicate the positions of the mutations in affected patients. Lane 1: 100-bp molecular weight marker. [file 1750-1172-5-33-S4.TIFF]

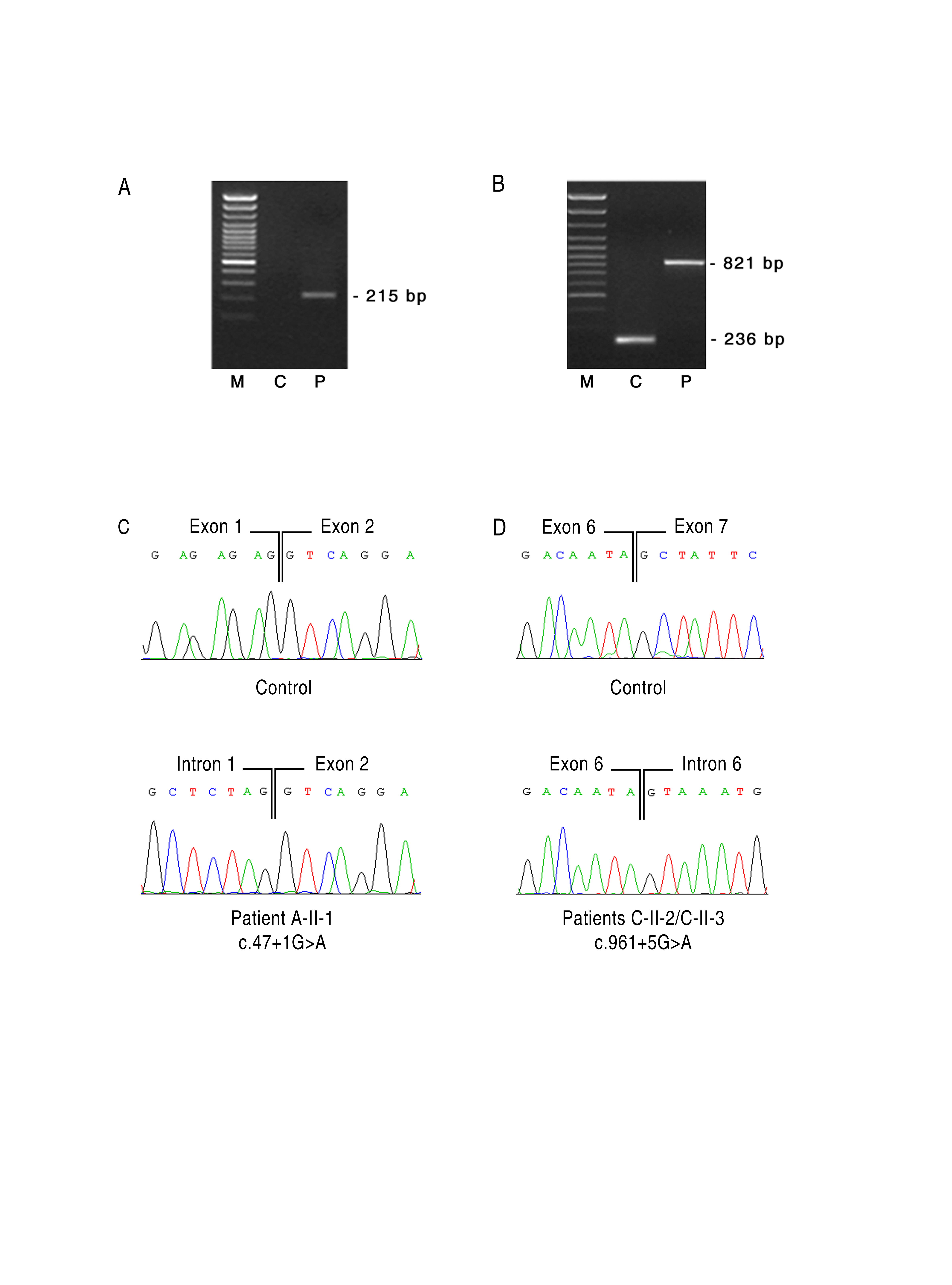

Supplement: Additonal file 5 — Supplementary Figure 4. Molecular characterization of the c.47+1G>A and c.960+5G>A ABHD5 mutations. A, RT-PCR of part of intron 1 and exon 2 from cDNA of the control subject (no amplification product) and A-II-1 patient (215 bp); Lane 1: 100-bp molecular weight marker. B, RT-PCR of exons 6 and 7 from cDNA of a control subject (236 bp) and C-II-2 or C-II-3 patient (821 bp); Lane 1: 100-bp molecular weight marker. C, Partial sequences of exon1/exon2 from cDNA of a control subject and of intron1/exon2 from cDNA of the A-II-1 patient. D, Partial sequences of exon6/7 from cDNA of a control subject and of exon6/intron6 from cDNA of the C-II-2 or C-II-3 patients. [file 1750-1172-5-33-S5.JPEG]

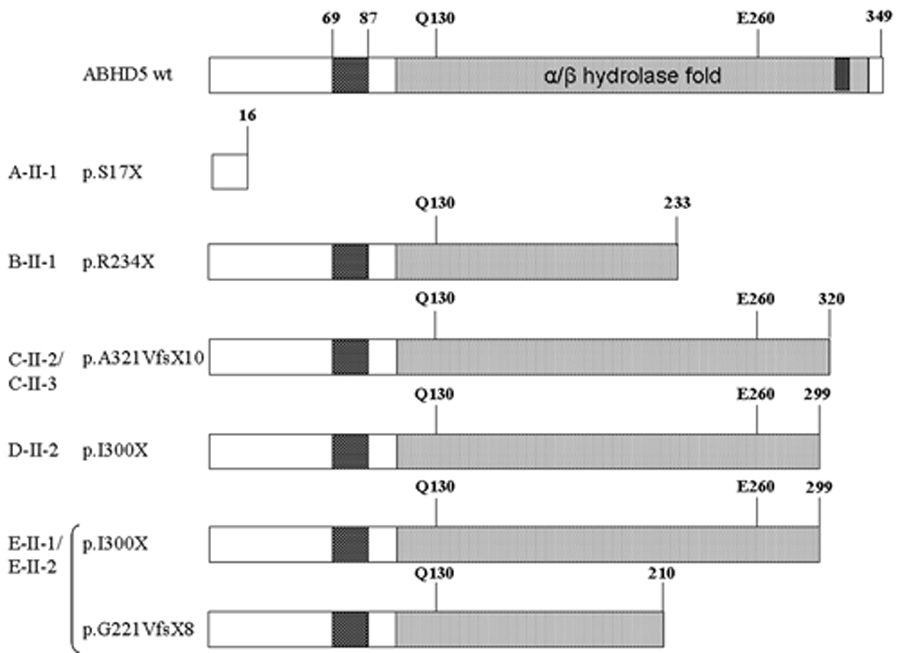

Supplement: Additional file 6 — Supplementary Figure 5. Domain organization of wild-type and mutant ABHD5 variants. The ABHD5 theoretical variants resulting from the six mutations identified in this study are truncated proteins lacking different portions of wild-type ABHD5. Five of the six mutant variants retained the hydrophobic motif, located between residues 69 and 87 (dark-grey area), that represents the putative lipid-binding domain. However, all six mutant proteins lacked the HX4D motif between amino acids 327 and 332, specific for proteins with acyltransferase activity. Q130 and E260, reported in the models, have previously been identified as essential residues for ABHD5-perilipin interaction and for ATGL activation. [file 1750-1172-5-33-S6.JPEG]
