# Supplementary material for: Clinical and genetic characterization of chanarin-dorfman syndrome patients: first report of large deletions in the ABHD5 gene
Source: Orphanet J Rare Dis. 2010 Dec 1;5:33. doi: 10.1186/1750-1172-5-33 (PMC3019207; doi:10.1186/1750-1172-5-33)
Supplement: Additional file 2 — Supplementary Table 1. Primers for genomic and cDNA analysis of ABHD5 gene. [file 1750-1172-5-33-S2.DOC]

**Supplementary Table 1**. Primers for genomic and cDNA analysis of ABHD5 gene

| Primer pairs | Oligonucleotide sequence | Primer location |
| --- | --- | --- |
| **Amplification of ABHD5 putative promoter** **from DNA** | aF 5’-AATATAAACACCCGTATGCAAATAAA-3’  aR 5’-TACTACAAATGGCTTTATGCCAAT-3’  bF 5’-GGCTAATATCCAGAATCTACAATG-3’  bR 5’-AACATTTGGGTTGGTTCCAAG-3’ | Promoter-region  “  “  “ |
| B. Amplification of ABHD5 gene from DNA | 1F 5’-TAAAACACCTAACTCATTCGGG-3’ | Promoter-region -313bp |
|  | 1R 5’-TTATACAACAACGGGGCGGAC-3’ | Intron 1 |
|  | 2F 5’-CCACCATGCTTTGTGCATGTTAG-3’ | Intron 1 |
|  | 2R 5’-AAACAAATCTCCTTGGGGTC-3’ | Intron 2 |
|  | 3F 5’-TGAGGTAGGTCTTCCCCTTT-3’ | Intron 2 |
|  | 3R 5’-AGAGAATGTCTGCCTTGTGG-3’ | Intron 3 |
|  | 4F 5’-CGTGAAGGTTTTTGAAGGT-3’ | Intron 3 |
|  | 4R 5’-GGGTTCAGGGTTTTCTTGTT-3’ | Intron 4 |
|  | 5F 5’-CACAGACAAGCACTAAAACTTTC-3’ | Intron 4 |
|  | 5R 5’-GACCTGGGGTCAGAAGTTCA-3’ | Intron 5 |
|  | 6F 5’-CTTAGGTGCTGGAAAAGCTA-3’ | Intron 5 |
|  | 6R 5’-GTAGTTCACGGTTTGGACAT-3’ | Intron 6 |
|  | 7F-5’-TTTAAATACAGTGGCTCTCACTT-3’ | Intron 6 |
|  | 7R 5’- TCAGAAATCACTTCCTAAATTGG-3’ | Intron 7 |
| C. Amplification of ABHD5 large deletions from DNA | 6F 5’-CTTAGGTGCTGGAAAAGCTA-3’ | Intron 5 |
|  | 7R 5’- TCAGAAATCACTTCCTAAATTGG-3’ | Intron 7 |
|  | 4aF 5’-atcatctcacaaatcagcgc | Intron 4 |
|  | 6R 5’-GTAGTTCACGGTTTGGACAT-3’ | Intron 6 |
| *D. Amplification of ABHD5 full-lenght cDNA* | 8F 5’-TGCGCCGCCTTAAGTGCCGC-3’ | Exon 1 |
|  | 8R 5’-TAAAGGGTTAAAGGGAGTCAATGCTGCTC-3’ | Exon 4 |
|  | 9f 5’-AATCATCTCATTTTAGTGGAGCC-3’ | Exon 4 |
|  | 9R 5’-CAGTCACCAGGTTTTCCCATC-3’ | Exon 7 |
| E. Amplification of ABHD5 aberrant cDNAs | 2F 5’-CCACCATGCTTTGTGCATGTTAG-3’ | Intron 1 |
|  | 2aR 5’-gtgtgatatagacgtagggcacc-3’ | Exon 2 |
|  | 9aF 5’-TGCACCCTGACATTCCAGTTTCA-3’ | Exon 6 |
|  | 9R 5’-CAGTCACCAGGTTTTCCCATC-3’ | Exon 7 |
